# Intrathoracal cholecystitis calculosa in a right-sided posttraumatic diaphragmatic hernia: a case report

**DOI:** 10.1186/1752-1947-7-89

**Published:** 2013-04-02

**Authors:** Laura L Tahiri, Afrim Tahiri, Rifat Bajrami, Shpresa Hasimja, Antigona Hasani

**Affiliations:** 1 University Clinical Center of Kosova, Clinic of Surgery, Rrethi i Spitalit pn, Prishtina, 10000, Republic of Kosova; 2 University Clinical Center of Kosova, Clinic of Anesthesiology, Rrethi i Spitalit pn, Prishtina, 10000, Republic of Kosova

## Abstract

**Introduction:**

Injuries of the diaphragm were first described in 1541 by Sennertus and the initial repair was performed by Riolfi in 1886. Posttraumatic diaphragmatic hernia in adults is usually caused by blunt trauma and may remain asymptomatic and undiagnosed for many years. Right-sided tears are significantly less likely than left-sided tears because of the protective effect of the liver. They are associated with high mortality and morbidity. The rupture of the right side of the diaphragm and the presence of an inflamed gallbladder in the thoracic cavity are uncommon.

**Case presentation:**

We present the case of a 57-year-old Albanian man with prolapses of his gallbladder and other abdominal organs into the thoracic cavity through the herniation of his right hemidiaphragm due to trauma. The diaphragmatic hernia and gallstones seen in the thorax computed tomography scan were diagnostic. The organs herniated to the thoracic cavity were placed back into the abdominal cavity, a cholecystectomy was performed and the defect in the diaphragm was repaired with a prolene mesh graft during the operation. The patient was discharged 10 days after the surgical procedure, and no complications were reported.

**Conclusion:**

Diaphragmatic hernia should be considered as a possible diagnosis in patients with respiratory disorders or unusual shadows in the thoracic region after recently sustained injury or with a history of injury. The prolapse of a gallbladder is rare. The symptoms are uncharacteristic and patients with this disease may remain without symptoms for a long period. Treatment is surgical.

## Introduction

Injuries of the diaphragm were first described in 1541 by Sennertus and the initial repair was performed by Riolfi in 1886 [1]. Posttraumatic diaphragmatic hernia in adults is usually caused by blunt trauma and may remain asymptomatic and undiagnosed for many years. We report a case of delayed presentation of a patient with a rupture on the right side of his diaphragm and herniation of his abdominal organs and gallbladder (with stones) into the thorax. The rupture of the right side of the diaphragm and the presence of an inflamed gallbladder in the thoracic cavity are uncommon.

## Case presentation

A 57-year-old Albanian man admitted to the emergency department presented with a 10-day history of right-sided epigastric and chest pain, together with a mild cough, distension of the abdomen, and constipation. The pain had been treated with analgesics. The patient had had a traffic accident seven years previously. After the accident, in the emergency room, he had undergone drainage to the right side of the thorax because of pleural effusion and the splinting of his upper arm due to a humerus fracture on the same side. An abdominal ultrasound examination, at that time, had confirmed no evidence of organ injury.

**Figure 1:**
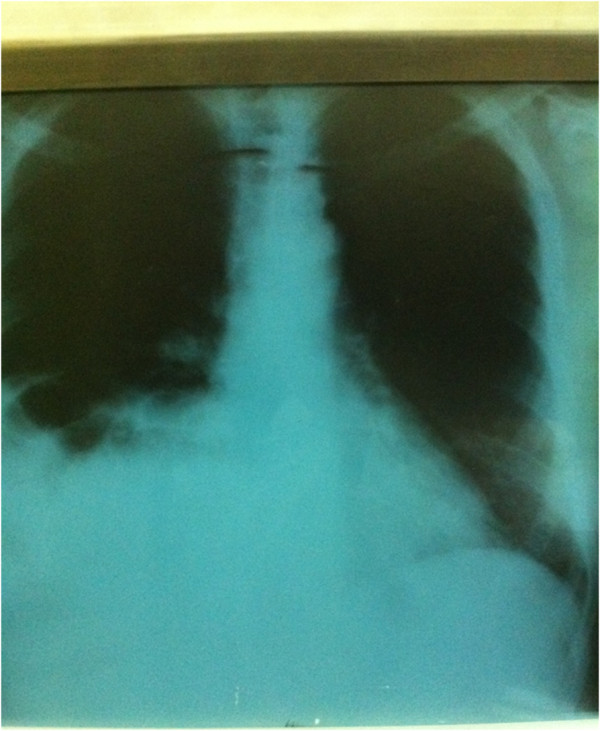
Chest X-ray showing elevation of the right hemidiaphragm.

On arrival at the emergency department, the presence of gallstones was suspected, but none of the preclinical results supported this diagnosis. He was afebrile; his abdomen was inflated and insensitive to palpation. All laboratory results were within normal limits except for leucocytosis. The initial chest X-ray showed elevation of the right hemidiaphragm, an infiltrative shadow in the basal lobe of his right lung, and multiple radiolucencies over his right hemithorax (Figure 1). The stones in the gallbladder were invisible. Black pigment or mixed gallstones may contain sufficient calcium to appear radiopaque on plain films. Only 15 to 20 percent of gallstones are visible on simple radiographs [2].

**Figure 2:**
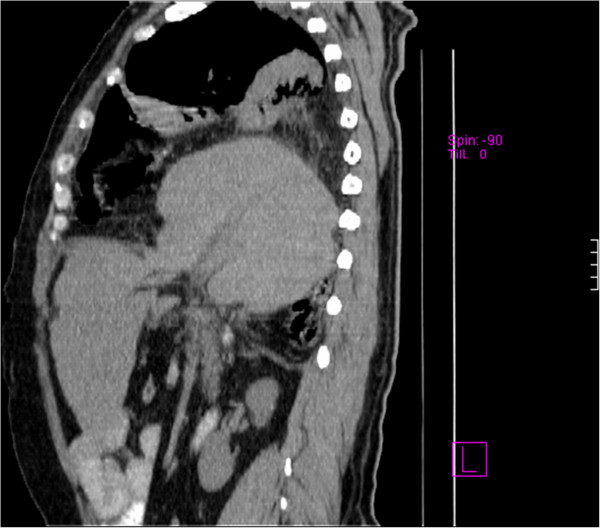
Computed tomography (CT) scan showing intrathoracal displacement of two-thirds of the liver.

**Figure 3:**
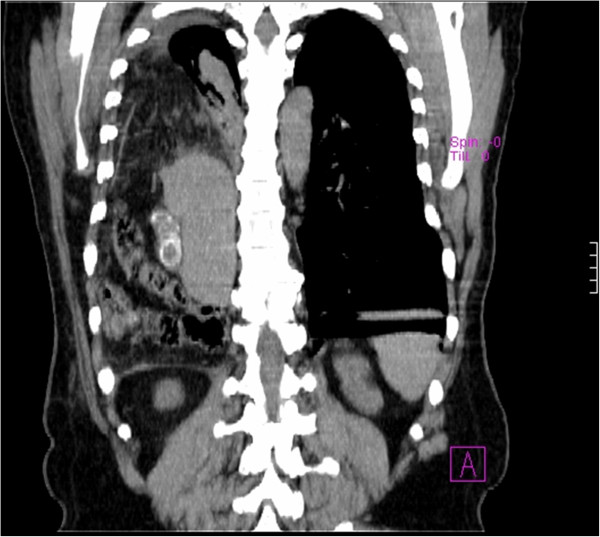
Computed tomography (CT) scan showing intrathoracal displacement of the cholecyst with three stones.

The definite diagnosis was made through a thoracic-abdominal computed tomography (CT) scan, which revealed intrathoracic displacement of his liver and part of the colon, and his gallbladder with regular walls and with three 17mm stones around its neck (Figures 2 and 3).

The patient underwent surgical intervention via an abdominal approach. Intraoperative findings confirmed that the omentum, colon, gallbladder and 70 percent of his liver had herniated into the thoracic cavity. The organs were placed back into the abdominal cavity. The omentum was dissected off its adhesions and retrieved. A cholecystectomy was performed, and three stones were found in the gallbladder. The defect in the diaphragm, measuring 13cmx8cm, was repaired with a prolene mesh graft. A right-sided chest tube was inserted for drainage. The patient remained stable during the intraoperative phase. Postoperative recovery was uneventful, and he was well at the follow-up. The chest drain was removed on the fifth day after the surgery. The patient was discharged 10 days after the surgical procedure without any problems.

## Discussion

Rupture of the diaphragm is a serious complication of blunt trauma. Diaphragmatic hernia is often initially overlooked in the acute setting and is only correctly diagnosed in 30 to 40 percent of cases [3-5]. In our case, diaphragmatic injury was diagnosed seven years after the injury.

A rupture of the right side of the diaphragm is uncommon. Approximately 69 percent of hernias are left-sided, 24 percent are right-sided, and 15 percent are bilateral [6]. Although autopsy studies have revealed equal incidence of right- and left-sided diaphragmatic ruptures, antemortem study reports suggest 88 to 95 percent of them occur to the left side [7-9]. Right-sided tears are significantly less likely than left-sided tears because of the protective effect of the liver. They are associated with high mortality and morbidity [10,11].

Prolapse of the gallbladder is rare. There are just a few articles that report the presence of the gallbladder in posttraumatic diaphragmatic hernia [12-17]. In our case, an impeded presentation of the ruptured diaphragm and the presence of the gallbladder can be explained by the hypothesis of delayed detection. The ‘delayed detection’ hypothesis assumes that a diaphragmatic defect created at the time of the injury becomes clinically evident only when herniation occurs or when problems arise from the hernia contents. In our case, the symptoms appeared with the presence of stones in the gallbladder [1,18].

The operative treatment for diaphragmatic rupture consists of hernia reduction, pleural drainage and repair of the defect [19]. The surgical approach could be either transabdominal or transthoracic, or both. However, laparoscopic surgery, thoracoscopy, or artificial patches have become very popular in recent years. Our patient underwent a transabdominal approach and a mesh repair of his diaphragmatic hernia. A transabdominal approach is recommended for a cholecystectomy, even if the gallbladder is in the thoracic cavity. On the other hand, the mesh decreased the risk of a recurrence of the hernias. This was caused by the shearing of a stretched membrane and avulsion of the diaphragm from its points of attachments due to a sudden increase in intra-abdominal pressure, transmitted through the viscera.

## Conclusions

Diaphragmatic hernia should be considered as a possible diagnosis in patients with respiratory disorders or unusual shadows in the thoracic region after recently sustained injury or with a history of injury. The prolapse of a gallbladder is rare. The symptoms are uncharacteristic and patients with this disease may remain without symptoms for a long period. Treatment is surgical.

## Consent

Written informed consent was obtained from the patient for publication of this case report and accompanying images. A copy of the written consent is available for review by the Editor-in-Chief of this journal.

## Competing interests

The authors declare that they have no competing interests.

## Authors’ contributions

AT and RB analyzed and interpreted the patient data regarding the diagnosis and performed the surgery. LT and AH were major contributors in writing the manuscript, SH performed the anesthesia and postoperative treatment. All authors read and approved the final manuscript.
